# Taeniasis Presenting as Motile Worms in the Stools: An Emerging but Neglected Parasitic Disease

**DOI:** 10.7759/cureus.30066

**Published:** 2022-10-08

**Authors:** Venkataramana Kandi, Vinay Kumar Moses

**Affiliations:** 1 Clinical Microbiology, Prathima Institute of Medical Sciences, Karimnagar, IND; 2 Microbiology, Rajiv Gandhi Institute of Medical Sciences, Adilabad, IND

**Keywords:** cysticercosis, zoonotic infection, reservoirs, food and water contamination, infestation, taenia saginata, taenia solium, taeniasis

## Abstract

Many parasitic infections essentially pose a significant public health problem throughout the world. However, the consequence of the issue is felt more among developing countries like India. This is majorly due to unsatisfactory hygienic practices and overcrowding, among others. Moreover, taeniasis caused by the parasites *Taenia solium *and *Taenia saginata *is a zoonotic infection transmitted via pigs and cattle, respectively. Even though the animals are the primary hosts where the parasite lives, humans also can suffer from infestation after consuming the eggs in contaminated food and water. Interestingly, there is a lower level of awareness regarding such types of *Taenia *infections in humans. On the contrary, most physicians and some people know that parasite transmission generally happens from the consumption of raw/undercooked pork and beef, which may result in a more serious consequence like neurocysticercosis. However, *Taenia *infestation in the intestines equally affects humans where people become reservoirs of infection, and suffer from severe morbidity. We present a case of worm infestation in a teenage girl who presented with severe peri-anal itching and motile worms in the stools.

## Introduction

According to an update by the Centers for Disease Control and Prevention (CDC), several parasitic diseases including the Chagas disease caused by *Trypanosoma cruzi*, toxoplasmosis caused by *Toxoplasma gondii*, and cysticercosis/taeniasis caused by *Taenia *species are being neglected. This could lead to their emergence and result in significant morbidity among the affected population [1]. *Taenia *species belong to the family *Taenidae *and the order *Cyclophyllidea*. They are called cestodes or tape-like worms, which are hermaphrodites (no separate male and female), and the worms are segmented and dorsoventrally compressed without having a body cavity. *Taenia solium *(*T*.* solium*) is commonly called a pork tapeworm, and *Taenia saginata *is a beef tapeworm. Taeniasis caused by *T*. *solium *generally results in cysticercosis. In this condition, people ingest the encysted larvae in undercooked/improperly cooked meat. After ingestion, and upon reaching the intestine, excystation ensues the release of larvae, which penetrate the intestinal wall. Later, the larvae gain access to blood vessels and travel through to reach various organs of the body including the brain and other tissues where they become encysted. However, the infection can also happen quite frequently through the consumption of food and water contaminated with eggs. During such entry, the eggs hatch out larvae known as oncospheres, which possess three pairs of hooklets. The oncosphere attaches to the intestinal walls using the hooklets, grows into an adult worm, and undergoes self-fertilization. The mature proglottids/segments lay eggs that are passed out on stools. However, in some people, the parasite segments are also passed out in the feces. In this case study, we report a worm infestation in which the patient presented with perianal itching and gave a history of passing worms in the stools. 

## Case presentation

**Figure 1:**
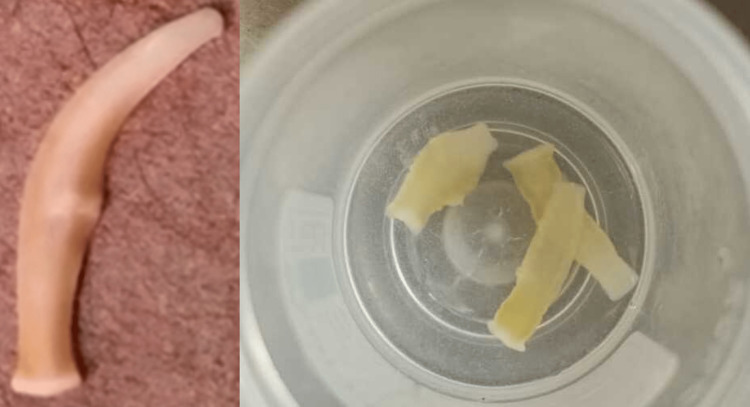
The worm segments noticed in the stool specimen

A 17-year-old girl presented to the outpatient clinic and complained about the passing of worms in the stools for the past 15 days. The patient was previously healthy and had no underlying diseases/co-morbidities. She belonged to a Muslim community and gave a history of consuming beef regularly and had never consumed pork. The patient also witnessed perianal itching. She did not notice any blood in the stools; however, she gave a history of the passage of worms with/or without any stool material. There was no history of abdominal pain, nausea, or vomiting. However, the patient felt mild abdominal discomfort occasionally. The patient revealed that there was a loss of appetite without any significant weight loss. She had a normal menstrual cycle and reported vaginal discharge. On clinical examination, the patient was noted to have a soft abdomen with no signs of abdominal lumps. Preliminary suspicion was enterobiasis based on the perianal itching reported by the patient. On the suspicion of worm infestation, a stool examination for parasites was advised. The routine microscopic stool wet mount did not reveal the presence of parasitic eggs. However, careful macroscopic examination showed the presence of worms that were ivory-colored as shown in Figure 1.

Some of the worms harvested from the stool specimen were noted to show motility as shown in Video 1

**Video 1:** Motile proglottid noticed in the stool specimen

Due to the mobility of the worm-like structure, the identity of the parasite was not clear. Therefore, the images and a video were sent to the diagnostic assistance of the CDC, Atlanta. The worm was identified as a *Taenia *proglottid segment, based on the elongated shape, motility, and unilateral genital pore. However, the species-level identity cannot be ascertained further without staining of the proglottid to determine the shape of the uterine branches. Recovery of the scolex/head can allow distinction, but finding the scolex is extremely rare since it hooks itself to the intestinal wall with the help of rostellum, suckers, and/or hooklets.

The patient was prescribed albendazole (400 mg once daily), ivermectin (6 mg twice daily), and doxycycline (100 mg twice daily) for three days. The patient was also advised to take multivitamins and calcium supplements and the patient and her parents were counseled about measures required to prevent future infections. A repeat stool examination was not possible since the patient did not return for a review despite the advice of the physician.

## Discussion

Taeniasis is a parasitic infection/infestation caused by *T*.* solium*, *T*. *saginata*, and *T*. *asiatica*. *T*. *solium, *and *T*. *asiatica *are acquired from the consumption of undercooked pork and *T*.* saginata *infections occur after eating undercooked beef. Most often than not, infection remains silent, asymptomatic, and uncomplicated. However, in some individuals, especially after consuming undercooked meat contaminated with cysticercus larva, or food and water contaminated with eggs of *T*.* solium*, cysticercosis may develop. This is defined as the presence of encysted larvae inside the muscle tissues including the brain and other organs [2]. According to the World Health Organization (WHO), up to 80% of the people who suffer from neurocysticercosis live in developing countries. This is majorly attributed to poor hygiene and the unavailability of safe drinking water [3]. Taeniasis may present as abdominal pain, nausea, diarrhea, or constipation. However, the symptoms may not be profound and the parasite can fully grow into an adult within the intestine roughly two months after consuming beef/pork containing encysted larvae. If left untreated, the parasite may die after about two to three years [3].

In the present case, the patient belonged to a Muslim community and had given a history of consumption of beef regularly and had never eaten pork. So, it may be assumed that the patient could have been infected by *T*.* saginata*. However, due to the absence of the head part of the parasite in the stool, confirmation of the same could not be achieved.

Although most infections of *Taenia *remain self-limited, some people may suffer from the consequences of dissemination. A Japanese man noticed several subcutaneous nodules along with the passage of worm segments in the stools. This patient was initially treated for intestinal taeniasis; however, after a thorough clinical and diagnostic work-up, the patient was diagnosed as suffering from disseminated cysticercosis [4]. This case report emphasizes the role of clinicians to identify the possible cases of autoinfection, cysticercosis, and dissemination of infection among those who suffer from intestinal worm infestation. 

Another recent case study of taeniasis has noted the persistence of the worms for a decade in the intestines despite treatment [5]. This report emphasizes the need for physicians to follow up with the patients during and after the prescribed therapy to confirm the elimination of the parasite.

Recently, small bowel obstruction due to taeniasis in a 65-year-old woman with co-morbidities like diabetes, and hypertension was reported. Here, the patient presented with acute abdomen and constipation [6]. This case reasserts the fact that the *Taenia *infestation in the intestines could lead to complications that may need surgical interventions. 

Infections of *T*. *saginata *causing intestinal obstruction, perforation, and necrosis were previously reported [7,8]. A history of postprandial abdominal discomfort and epigastric pains for the past six years were noted [7]. It is imperative to know that the parasite can survive in the intestines for an increased amount of time unlike two to three years as previously known.

A case of severely inflamed gallbladder diagnosed as a case of cholecystitis was recently reported in a 70-year-old patient who presented with vomiting and colicky abdominal pain. Laparoscopic surgical drainage of the gall bladder revealed a parasite-like structure, which was later identified as *T*. *solium *[9]. 

The parasitic eggs may not be present in the stools. Identity of the worm segments is difficult and requires experienced microscopists. The diagnosis through molecular methods may be helpful in cases with unclear morphological characteristics such as immature proglottids. Moreover, antigen-based enzyme-linked immunosorbent assay (ELISA) is particularly helpful to screen for infections in endemic regions [10,11].

## Conclusions

Taeniasis is an intestinal infection caused by *Taenia *species. Most infestations remain undiagnosed owing to mild symptoms including abdominal discomfort and perianal itching caused by motile worms. In some people, the infection can stay asymptomatic. Moreover, infections are generally self-reported wherein the patients complain of noticing worms in the stools. Routine stool examination may be negative for parasitic eggs due to immature parasite forms. However, the parasite may cause complications like intestinal obstruction, perforation, and cholecystitis among others. The long-term persistence of infestation may predispose people to autoinfection, malnutrition, parasite dissemination, and cysticercosis. Early diagnosis and parasite elimination may contribute to lowering long-term complications and improving the quality of life. 
